# Can Hip-Knee Line Angle Distinguish the Size of Pelvic Incidence?—Development of Quick Noninvasive Assessment Tool for Pelvic Incidence Classification

**DOI:** 10.3390/ijerph19031387

**Published:** 2022-01-26

**Authors:** Shota Yamada, Takeshi Ebara, Toru Uehara, Taro Matsuki, Shingo Kimura, Yuya Satsukawa, Akira Yoshihara, Kazuji Aoki, Atsushi Inada, Michihiro Kamijima

**Affiliations:** 1 Department of Occupational and Environmental Health, Graduate School of Medical Sciences, Nagoya City University, Nagoya 4678601, Japan; ptyamada@med.nagoya-cu.ac.jp (S.Y.); tmatsuki@med.nagoya-cu.ac.jp (T.M.); kamijima@med.nagoya-cu.ac.jp (M.K.); 2 Department of Rehabilitation, Nagoya City University West Medical Center, Nagoya 4628508, Japan; toru8toru22@gmail.com (T.U.); kimura.shingo.1234@gmail.com (S.K.); satsu.rh@gmail.com (Y.S.); football_4218@yahoo.co.jp (A.Y.); 3 Faculty of Rehabilitation Sciences, Nagoya Gakuin University, Nagoya 4568612, Japan; aokikazu@ngu.ac.jp; 4 Spine Center, Department of Orthopedic Surgery, Nagoya City University West Medical Center, Nagoya 4628508, Japan; a.inada.24@west-med.jp

**Keywords:** pelvic incidence, low back pain, hip–knee line, anthropometry, ROC curve, reliability

## Abstract

This study aimed to explore effective measurement angles for pelvic incidence (PI) classification and to develop a quick, noninvasive assessment tool for PI classification. We defined five variation types of hip–knee line (HKL) angles and tested the discrimination ability of the receiver operating characteristic (ROC) analysis using 125 photographs of upright standing posture from the right lateral side. ROC analysis revealed an applicable HKL angle defined by the line connecting the most raised part of the buttock and the central point of the knee and the midthigh line. The acceptable cut-off points for discriminating small or large PIs in terms of HKL angle were 18.5° for small PI (sensitivity, 0.91; specificity, 0.79) and 21.5° for large PI discrimination (sensitivity, 0.74; specificity, 0.72). In addition, we devised a quick noninvasive assessment tool for PI classification using the cut-offs of the HKL angle with a view to practical application. The results of intra- and inter-rater reliability ensured a substantial/moderate level of the tool (Cohen’s kappa coefficient, 0.79; Fleiss’s kappa coefficient, 0.50–0.54). These results revealed that the HKL angle can distinguish the size of the PI with a high/moderate discrimination ability. Furthermore, the tool indicated acceptable inter-/intra-rater reliability for practical applications.

## 1. Introduction

Low back pain (LBP) is one of the most common health problems worldwide. According to a systematic review, the lifetime prevalence in the general population is 60–80% [1]. The one-year prevalence in the general population is 38%, the one-month prevalence is 23–26%, and the point prevalence is 12–14% [2,3,4]. According to the Global Burden of Diseases study, the average disability-adjusted life years (DALY) for all generations of LBP increased from 1.7 years to 2.5 years as of 2019 compared to 1990 [5]. Out of 310 diseases, LBP was the leading cause of the years lived with disability in 2017 [6]. Furthermore, the DALY of LBP in the working generation is at a high level of 3.9 years. Hence, LBP, the most commonly reported work-related musculoskeletal disorder, has also received attention in terms of outcomes leading to a decline in labor productivity and economic loss [7,8].

Effective measures for LBP are still controversial. Although a recent meta-analysis [9] showed some intervention effects such as exercise in combination with education, there is no solid evidence found for others such as back belts, ergonomic adjustments, or training on manual material handling [10]. One of the possible reasons for such a discrepancy in the effect of LBP can be found in nonpersonalized countermeasures without considering individual biomechanical features of the lumbar spine.

For example, pelvic incidence (PI), which is measured by lateral spine radiography, has been attracting attention as a determinant of LBP. PI is an important clinical parameter generally applied in the field of spinal surgery after Legaye et al. reported it in 1993 [11]. Since then, it has been used for PI-based spinal surgery alignment design and analysis of clinical outcomes [12,13]. PI has some features representing an angle that: (1) defines the lumbar lordosis [14,15], (2) indicates the inclination of the sacrum in the pelvis [16], (3) is fixed during puberty [17], and (4) is a constant independent of posture changes [18]. Recent research shows that PI also has no differences in any age groups and sex [19], but there are differences in ethnicity [20]. PI represents the physiological lordosis of the lumbar spine, inducing differences in mechanical stress on the intervertebral discs and facet joints depending on the degree of angle. If we could successfully grasp the angle of PI in a simple way without exposure to X-rays, PI may be available as a personalized preventive measure for LBP according to the angle of individual PIs. Thus, we devised a noninvasive indirect measurement using surrogate indicators to estimate the PI using anthropometric landmarks on the body surface. Our previous studies [21,22] revealed a surrogate angle on the body surface reflecting the PI. The surrogate angle of the PI can be defined as “the angle between the line connecting the upper edges of the greater trochanter and iliac crest, and the line connecting the upper edge of the iliac crest and the buttock at the same height as the greater trochanter”. It has sufficient practical reliability to estimate the PI (R^2^ = 0.63), although the measurement method requires substantial time (about 15 min/person) and physiotherapist skill for palpation. Another possible approach that can more easily determine the classification of PI might be to use a body silhouette. As shown in a commentary paper by Ramchandran et al. providing a theoretical interpretation of PI, changing PI visually reflects the waistline and buttocks due to increasing pelvic overhang with increasing sacroiliac joint angulation [23]. For further simplification, we can hypothesize that those with an anatomically large PI will have raised buttocks; in contrast, those with a small PI will have a smaller bulge in the buttocks. However, little research has been conducted to determine whether the thickness of the buttocks (silhouette) may reflect the actual size of the PI.

Therefore, we focused on the thickness of the buttocks on the silhouette to find an easy, noninvasive way to classify the stage of the PIs. The purpose of this study was to explore effective measurement angles for PI classification in terms of discrimination ability using receiver operating characteristic (ROC) curve analysis, and to identify the availability of visual buttock silhouettes (Study 1). Furthermore, the intra- and inter-rater reliability of a devised tool for simple PI classification focusing on the visual buttock silhouette was assessed (Study 2).

## 2. Materials and Methods (Study 1: Exploring Effective Surrogate Angles for PI Classification Focusing on the Buttocks)

### 2.1. Measurement Angular Definition

We discussed potential landmarks applicable for the estimation of PI based on anatomical, anthropometric, and physiotherapy aspects: (1) the most raised part of the buttock; (2) top of the head; (3) the anterior (patella), central, and posterior points of the sagittal knee; and (4) center of the thigh (the anterior–posterior diameter of the transition between the buttocks and the thigh). To verify the PI with the appearance of the body silhouette, the posterior and anterior surfaces of the knee are landmarks that can be easily confirmed, and have been used in many studies in ergonomics and clinical practice. Furthermore, the lines at the center of the knee and the center of the thigh on the lateral side (sagittal plane) are used to measure the range of motion of the hip and knee joints in clinical situations. The parietal point was adopted as a frequently used landmark for cervical and standing posture analyses.

**Figure 1:**
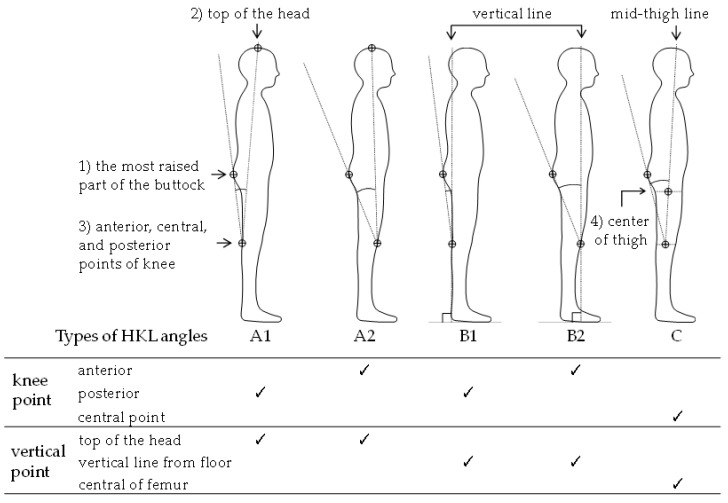
Five variation types of hip-knee line (HKL) angles.

The hip–knee line (HKL) angles were defined as the angle between the following two intersecting lines on the sagittal plane: the line connecting the most raised part of the buttock and either the anterior, central, or posterior knee; and the line connecting either knee point and either of vertical lines as the parietal, vertical, or the midthigh line. The midthigh line was defined as the femoral axis (the central part of the anterior–posterior diameter of the knee and the center of the thigh). As a combination of the lines, five variation types of HKL angles (A1, A2, B1, B2, and C) were defined as shown in Figure 1.

### 2.2. Procedures

The 125 voluntary participants (71 males, 54 females; average age 55.9 ± 18.9 years) were recruited in line with the following inclusion criteria: (1) normal spinal alignment with sagittal vertical axis less than 40mm, (2) standing alignment without knee flexion contracture, (3) no difference in leg length, and (4) maintained postural standing stability. We provided a sufficient explanation for this study (IRB No. 60-21-0072, Nagoya City University). They were then asked to assume a neutral posture in an upright position. Subsequently, a photograph of their upright standing posture from the right lateral side was taken using a digital camera (Power Shot S5, Canon Inc., Tokyo, Japan) fixed on a camera tripod at a height of 60 cm, with the focus distance to the subject set to 3 m.

### 2.3. Measurement of HKL Angles and Outcome Variable

The landmarks in each picture were obtained using the ImageJ ver. 1.48 software (NIH, Bethesda, MD, USA), and five types of HKL angles (A1, A2, B1, B2, and C) were measured using the landmarks.

The surrogate angle correlating with PI [21,22] was used to measure the outcome. The surrogate angle of the PI can be defined as “the angle between the line connecting the upper edges of the greater trochanter and iliac crest, and the line connecting the upper edge of the iliac crest and the buttock at the same height as the greater trochanter”. The details of the measurement and reliability of the landmarks used to estimate the surrogate angle by palpation have been described elsewhere [22]. The surrogate angle has sufficient practical reliability for estimating PI (R^2^ = 0.63).

### 2.4. Data Analysis and Statistical Analyses

The surrogate angles of PI obtained from the 125 photographs were classified by using quartiles: small (“S”, less than 42°, indicating the first quartile), medium (“M”, between 42° and 51°, the first and third quartile range), or large (“L”, more than 51°, the third quartile). To explore effective measurement angles for PI classification in terms of discrimination ability using ROC analysis, the outcome variables were further converted to the two dichotomous variables determining S/ML and SM/L.

To assess the discriminating ability of the HKL angles relative to S/ML and SM/L of PI, ROC curves were created by plotting values of sensitivity for the y-axis and 1-specificity for the x-axis to calculate the area under the curve (AUC). Discriminating ability was assessed by the AUC of the ROC curve by the following criteria: 0.5–0.7 as low accuracy, 0.7–0.9 as moderate accuracy, and >0.9 as high accuracy. We considered the HKL angle with the highest AUC out of the five HKL angles as a candidate angle applicable for a practical PI classification tool using the thickness of the buttocks. Focusing on the candidate angle with the highest AUC, the Youden index of the candidate angle, indicating the maximum difference between sensitivity and specificity, was calculated to assess the optimal threshold value (cut-off point) of S/ML and SM/L for HKL angles. These analyses were conducted using SPSS version 26.0 (IBM Corp., Armonk, NY, USA).

### 2.5. Criteria for Selecting Cut-Off Points Applicable to Practical PI Classification Tools Using Buttock Thickness

To devise a practical PI classification tool, we empirically considered that at least the following levels were needed as an acceptable range of cut-off points: ≥0.7 sensitivity and specificity. The acceptable range should include the maximum value of the Youden index. Furthermore, to ensure the availability of visual buttock silhouettes, angular differences between thresholds of S/ML and SM/L for PI classification should be set at least more than 3°.

## 3. Results (Study 1)

### 3.1. AUCs of HKL Angles Discriminating Small or Large PIs

**Figure 2:**
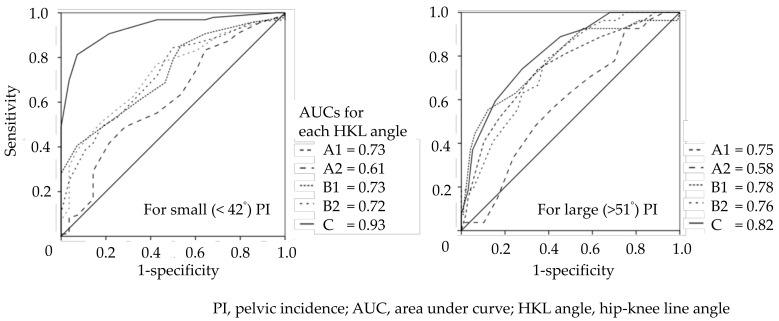
AUCs and HKL angles discriminating small (<42°) or large (>51°) PIs.

The average value of surrogate angles of PI was 47.0 ± 6.2° (male 45.4 ± 5.1°; female, 49.1 ± 7.0°) and BMI was 23.4 ± 3.4kg/m^2^. Figure 2 shows the AUCs of the HKL angles discriminating small (<42°) or large (>51°) PIs. The HKL angle with the highest AUC value was the HKL angle C, reaching a high accuracy of 0.93 for the small and moderate PIs, and 0.82 for the large PIs. On the other hand, the HKL angle with the lowest AUC was A2, which had low accuracies of 0.61 for the small PIs and 0.58 for the large PIs.

### 3.2. Cut-Off Points Applicable to Practical PI Classification Tool Using the Thickness of the Buttocks

In terms of the AUC value, the HKL angle C was considered to be an appropriate candidate for a practical PI classification tool using the thickness of the buttocks. Table 1 shows the results of the sensitivity, specificity, and Youden index of the HKL angle C. Columns that are shaded in gray in the table mean out of criteria (unacceptable range of cut-off points); namely, sensitivity and specificity of >0.7. The acceptable cut-off angles relative to S/ML discrimination ranged from 18.5° to 19.5° based on the criteria for selecting the cut-off points. The acceptable range also included a maximum Youden index value of 0.74 at 19.5°. Similar trends were observed in the strata of males and females.

The acceptable cut-off angle for SM/L discrimination was 21.5°, with a maximum Youden index value of 0.46. For male participants, the acceptable range was from 21.5° to 22.5°, while no acceptable range was found for female participants.

### 3.3. Devised PI Classification Tool Using the HKL Angle

**Figure 3:**
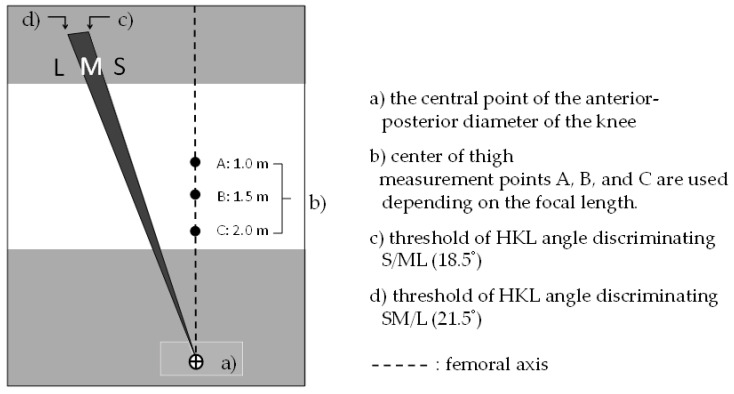
Practical PI classification tool using the HKL angle.

Emphasizing the ease of use and quick assessment, we developed a practical PI classification tool using the HKL angle, focusing on the thickness of the buttocks (Figure 3). The tool, which was printed on a transparent film, can be used to estimate the HKL angle sizes of S, M, and L by looking through it at the standing posture of the lateral side. Based on the acceptable range of cut-off points, the thresholds were set to 18.5° for S/ML and 21.5° for SM/L discrimination. In terms of the Youden index, the optimal cut-off angle was 19.5°; however, we applied an acceptable cut-off angle of 18.5° relative to S/ML. Angular differences between thresholds of S/ML and SM/L for PI classification should be set at least more than 3° in order to ensure the availability of visual buttock silhouettes. The tool enables quick assessment following two steps. First, align the points of (a) the central point of the anterior–posterior diameter of the knee and (b) the center of the thigh in the tool with those on the body surface of the subject. Depending on the focal length, measurement points A, B, and C were selected. Then, we determined where the most-raised point of the buttock was placed in the S, M, or L areas of the HKL angle.

## 4. Materials and Methods (Study 2: Assessing Intra-/Inter-Rater Reliability of the PI Classification Tool Using the HKL Angle)

### 4.1. Participants

A total of 14 healthy persons (working/studying at university/hospital, mean age 37.3 ± 10.0 years, 7 males and 7 females) voluntarily participated in this study as examiners for evaluating intra- and inter-rater consistency. Informed consent was obtained from the participants after an explanation of the study.

### 4.2. Materials Used for the Assessment

Of the 125 photos used in Survey 1, three sets of 24 photos were randomly selected for testing. Of these test materials, one was intended for use in prior training, and the other two were used for the assessment of intra-and inter-rater reliability. The ratio of classification was 1:2:1 for S:M:L. The developed PI classification tool using the HKL angle (Figure 3) was printed on a transparent film with a length of 14.8 cm and width of 21 cm (A5 size).

### 4.3. Procedures

The photos of the material were randomly projected on the screen and reduced to 1/2 of the actual subject size. This means that the focal distance between the examiner and the screen needed to be converted to 1/2 size. Therefore, the examiner was requested to sit in front of the screen 75 cm away at a focal distance of 1.5 m. Subsequently, the examiners were trained in advance on how to use the tool using the photo material for prior training.

**Figure 4:**
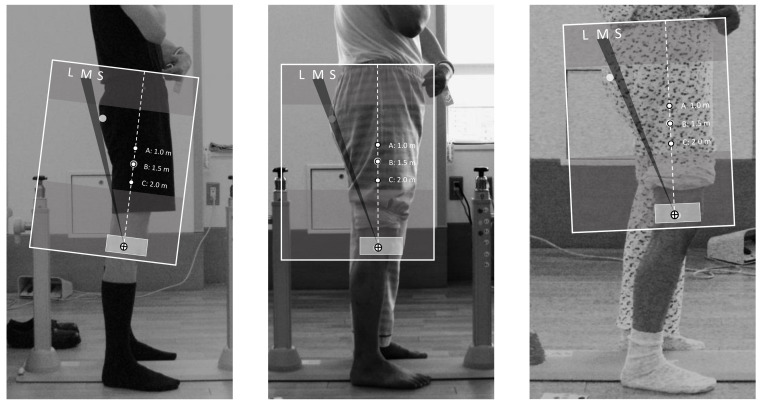
Application examples of the practical PI classification tool. Left photo shows in case of HKL angle classified “S”, meaning small (<42°) PI. Similarly, the center photo shows in case of the HKL angle classified “M”, meaning medium (42–51°) PI. The right photo shows in case of the HKL angle classified “L”, meaning large (>51°) PI.

In the training session, the examiners sitting in front of the screen were instructed to hold the tool and keep their eye level horizontal with the height of the subject on the screen. In addition, they were asked to place their eyes and the tool on a vertical line to the screen. After the setting, the researchers instructed them by showing 24 prior training photos one by one on how to use the tool already represented in Section 4.3. Examples of applications of the tool for practice are shown in Figure 4. Each examiner evaluated the materials for the assessment twice, with intervals for rest after training.

### 4.4. Data Analysis and Statistical Analysis

As one of the usability metrics, we measured the required time of first and second sessions to complete the classification of PI. The mean time (s/photo) of classifying 24 photos in each session was calculated. Furthermore, the correct rate was calculated as an effectiveness metric. The correct rate represented the extent to which examiners’ responses agreed with an external criterion for the classification of the HKL angle. We applied the surrogate angle of the PI as an external criterion, calculated in Study 1. A paired *t*-test was conducted to show the significant differences in the means for each metric between sessions.

Cohen’s kappa coefficient was calculated to examine the intra-rater reliability of measurements, showing the test–retest reliability of each examiner within the first and second sessions. Fleiss’s kappa coefficient (quadratic) was applied as a weighted inter-rater reliability, meaning reliability between the initial test (first session) and retest (second session) measurements obtained from all examiners. Both coefficients were estimated using 95% confidence intervals (CIs). According to Landis’s criteria [24], reliability levels of kappa coefficients were defined as 0 for poor, 0.01–0.20 for slight, 0.21–0.40 for fair, 0.41–0.60 for moderate, 0.61–0.80 for substantial, and 0.81–1.00 for almost perfect. These analyses were conducted using SPSS version 26.0 (IBM Corp., Armonk, NY, USA).

## 5. Results (Study 2)

### 5.1. Usability Metrics

The mean time (sec/photo) was 17.0 ± 4.7 for the first session and 13.6 ± 2.9 for the second session (Table 2); this difference was significant (mean difference, 3.4; 95% CI, 1.6–5.2; *t*(13) = 4.1, *p* = 0.001). The correct rate (%) reached 77.1 ± 6.9 for the first session and 79.8 ± 6.9 for the second session; no significant difference was found (*t*(13) = 1.0, *p* = 0.32).

### 5.2. Intra-/Inter-Rater Reliability

Table 2 also shows the results of intra- and inter-rater reliability of the PI classification tool using the HKL angle. The mean Cohen’s kappa coefficient as intra-rater reliability was 0.79 (95% CI, 0.61–0.96), indicating “substantial” levels of test–retest consistency. No sex difference was found in intra-rater reliability, even when focusing on the differences in the stratified analysis of the male and female subjects in the photos.

The mean Fleiss’s kappa coefficient as inter-rater reliability was 0.50 (95% CI, 0.47–0.53) for the first session and 0.54 (95% CI, 0.51–0.57) for the second session, both indicating “moderate” levels of consistency between examiners. We also conducted a stratified analysis, showing that raters tended to do better with female subjects than male ones (first session, 0.43 for males and 0.56 for females; second session, 0.50 for males and 0.57 for females, respectively).

## 6. Discussion

This was the first study to show that the HKL angle formed by lateral body landmarks focusing on the area of the femur and buttock can discriminate the classification of small or large PIs. We examined the optimal cut-off points for discriminating small or large PIs in terms of HKL angle, leading to an acceptable cut-off angle of 18.5° for S/ML and 21.5° for SM/L discrimination, considering a threshold range of more than 3° or larger angle. In addition, we devised a quick noninvasive assessment tool for PI classification using the cut-offs of the HKL angle with a view to practical application. The results of intra- and inter-rater reliability indicated certain limitations, but ensured a substantial/moderate level of the tool, meaning that it has sufficient potential for practical use. We will discuss the relationship between the HKL angle and PI, as well as the intra-and inter-rater reliability of the tool, in the following sections.

### 6.1. Relationship between the HKL Angle and PI Using the Visual Buttock Silhouette

HKL angle C reached a high accuracy of 0.93 for the small and moderate of 0.82 for the large PIs, whereas the HKL angle A2 showed the lowest AUC among all types of HKL angles. This difference deserves careful attention when interpreting the rationale for the HKL angles reflecting PI.

The HKL angles A1 and A2 shown in Figure 1 used the line connecting the top of the head and anterior/posterior knee points. The line was intended to reflect the alignment of the whole body, because whole-body alignment is a key clinical parameter generally applied in the field of spinal surgery to evaluate the bending characteristics of the spinal column from the standing posture of the sagittal plane. In addition, the Kendall classification [25], which is known as a representative of the classification of whole-body alignment, can be generally used to study muscle shortening and weakness in the clinical field of physiotherapy, and to correlate the relationship between the type of Kendall posture and lumbar pain [26]. Furthermore, PI is anatomically considered to have tendencies to be larger in the order of flat-back (S), sway-back (M), and ideal (L) and kyphosis–lordosis (LL) in the Kendall postures [27].

On the other hand, whole-body alignment maintains a standing posture under the influence of the movement control of many joints, such as the large angular displacement of the hip joint in a static standing position and curvature of the spinal column [28]. Moreover, the spinal column alignment compensates to maintain the whole-body balance of the head, chest, lumbar region, and lower limbs [29]. These findings provide an insight that the use of the line connecting the top of the head and anterior/posterior knee points for estimating PI, which is a part of spinal alignment indicating the inclination of the sacrum in the pelvis, might have disadvantages. This might also be explained by the differences between the HKL angles A and B. In fact, the HKL angles B1/B2 were intended to exclude the effect of whole-body alignment by using the line connecting the reference vertical line and the knee points. In addition, the HKL angle B1/B2 formed by the line connecting the raised part of the buttocks and either the anterior/posterior knee point or the reference vertical line can be considered to be an angle that simply indicates the degree of the uplift of the buttocks (sacrum). The AUCs of HKL angle B1/B2, excluding body alignment effects, were relatively high compared to HKL angle A2, regardless of the anterior/posterior knee positions. Moreover, the effect of compensation on body alignment is expected to be smaller for A1 than A2, owing to a relatively smaller angle, and leading to a relatively high AUC of A1 compared to that of A2. Therefore, it seems reasonable to suppose that HKL angle A2 showed a relatively low discriminating ability of PI due to these findings.

What needs to be emphasized here is that the HKL angle C focuses not on the whole spinal alignment, but on the limited area of the femur and buttock. It should be noted that HKL angle C defines an angle using a line connecting the central point of the anterior–posterior diameter of the transition between the buttocks and the thigh and the midpoint of the lateral joint space of the knee. This indicates the equivalent line connecting the great trochanter and the epicondyle of the femur used in the measurement of the flexion/extension angle of the hip and knee joint defined in the range of motion, or the line connecting the femoral epicondyle and the hip joint center of rotation defined in the International Society of Biomechanics’ recommendation [30].

This equivalent line has the following advantages: First, humans have a strategy of maintaining standing balance, the so-called “cone of economy” with the feet as the fulcrum; in other words, standing posture can be maintained in the conical space with the foot as the apex [31]. To compensate for changes in spinal alignment, the central line of the femur plays an important role in a stable standing posture. Humans compensate for age-related changes in spinal alignment by increasing posterior pelvic tilt and changing leg alignments to maintain the gravity line over the feet [32]. This indicates the possibility that the central line of the femur sensitively represents the extent of the PI. Second, according to a recent study that investigated the correlation between the sway of the center of gravity and spinal alignment such as PI, the larger the PI, the more the center of gravity moved forward [33]. This indicated that the axis of the thigh, when viewed from the lateral side, had shifted forward, and the larger the PI, the larger the angle of the HKL angle C. Thus, the HKL angle C reflecting the central line of the femur may be linked to postural fluctuations due to anterior–posterior bending of the body, which might have resulted in the highest AUC of HKL angle C sensitively reflecting PI.

### 6.2. Optimal Cut-Off Points Applicable to Practical PI Classification Tool

The acceptable cut-off values of the HKL angle C were 18.5° (sensitivity, 0.91; specificity, 0.79) for S/ML and 21.5° (sensitivity, 0.74; specificity, 0.72) for SM/L discrimination. Although it seemed to be sufficient for discriminating abilities in terms of application to a quick, noninvasive tool in practical use, some challenges remain. 

The first thing we noticed was that Youden index value at 18.5° of the HKL angle for discriminating small (<42°) PI showed 0.69, whereas the value for large (>51°) PI was as low as 0.46 at 21.5° of the HKL angle. One of the reasons for this is the PI characteristics of the Japanese population. Asians such as Japanese or Koreans generally have smaller PI values than Caucasians; for example, Kanemura reported Japanese PI as 46.7 ± 8.7° [34], and another similar study showed 46.7 ± 8.9° in 86 Japanese healthy adults aged from 23 to 59 years [35], and 47.8 ± 9.3° for the Korean population [36]. In contrast, 52.0 ± 9.0° was reported from the United States [12], 50.6 ± 10.2° [37] and 54.7 ± 10.6° [38] from France, and 50.2 ± 10.0° [11] from Belgium. Thus, the Japanese population not only has a smaller PI, but also a narrower range of standard deviation than Caucasians. Another reason might be the feature of the Japanese physique with relatively flat, smaller swelling of the buttocks compared to Westerners [35]. This means that even if the PI was large, the HKL angle tended to be underestimated in the case of focusing on the visual buttock silhouette.

Second, we should focus on the differences in the stratified analysis of male and female participants. The Youden index is relatively smaller in females than in males, indicating that the HKL angle might have disadvantages when applied to female patients. This might be due to the anatomical sex difference in the pelvis. The female pelvis has a large lateral diameter and is circular, and the anterior–posterior direction of the female is also larger than that of the male [39]. In this study, since we defined and assessed the HKL angle from the lateral side, such an anatomical feature may have biased the HKL angle in female participants. Another possibility may depend on the sample population used in this study. In general, it is believed that there is no sex difference in PI itself, although the samples of participants included in this study had a slight tendency to have a larger PI of 49.1 ± 7.0° in females, as compared to 45.4 ± 5.1° for males. The PI obtained from the 125 photographs were classified by quartiles regardless of sex, and the large PI was tentatively defined as >51° using the threshold in the third quartile. The large PI category consequently resulted in more females than males. Such biases might have led to a relatively low discriminating ability under the condition of SM/L.

### 6.3. Intra-/Inter-Rater Reliability of Tool Using HKL Angle

As shown in Table 2, the mean Cohen’s kappa coefficient as intra-rater reliability was 0.79 (95% CI, 0.61–0.96), indicating “substantial” levels of test–retest consistency. No sex difference was found in intra-rater reliability, even when focusing on the differences in the stratified analysis of the male and female subjects in the photos. 

In contrast, some challenges seem to be in the inter-rater reliability in the results of Fleiss’s kappa coefficients, which might be considered insufficient levels due to “moderate” consistency between examiners. However, it should be noted that Fleiss’s kappa coefficient contains a paradox, meaning that the kappa statistic may behave inconsistently in case of strong agreement between raters, since this index assumes lower values than would have been expected [40]. Second, the stratified analysis showed tendencies to be better for female subjects than male subjects (first session, 0.43 for males and 0.56 for females; second session, 0.50 for males and 0.57 for females). This might be related to the points previously discussed regarding the anatomical sex difference of the pelvis and sampling biases resulting in the inclusion of more females in the large PI category. Third, we should focus on improving the inter-rater reliability in the second session compared to the first session. We provided sufficient training to examiners using the 24 prior training photos individually. Nevertheless, we observed a tendency to be better in the 2nd session. Furthermore, a significant improvement in the meantime was observed between sessions. These results indicated that they did not reach a plateau, so further improvements in the inter-rater reliability could be expected.

Considering the results comprehensively, the devised assessment tool for PI classification using the cut-offs of the HKL angle has sufficient potential for practical use.

### 6.4. Practical Implication and Limitation

This study provides useful information about how to estimate PI using the HKL angle in a validated, noninvasive way. PI is considered to be one of the possible determinants of low back pain, and has been attracting attention as a new countermeasure considering individual biomechanical features of the lumbar spine. This knowledge can contribute not only to improving the new insight of occupational health research, but also to developing new noninvasive technologies for measuring PI using image recognition with artificial intelligence.

This study had some limitations. First, the values of PI were estimated by using the surrogate angle, not measured by X-rays. The surrogate angle has sufficient reliability for estimating PI under practical use (R^2^ = 0.63); however, the remaining variation of R^2^ can lead to misclassification into small, medium, or large PIs. Second, the study population was limited to Japanese adults, so the proposed acceptable range of cut-off points might vary with ethnicity [41]. Third, the small sample size may lead to imbalanced distributions of the target variable so that the sample size would need to be even larger in terms of the robustness of the results [42]. Fourth, a morbidly obese patient and an underweight patient with the same PI will likely have a different HKL angle; however, this study could not provide any evidence of the effect of BMI on the HKL angles. Japanese have a relatively low BMI compared to other ethnicities; morbidly obese patients (BMI > 35) are rare in the Japanese population, and none were in the current study population. In addition, there were only five underweight participants (BMI < 18.5). Further research is warranted in various populations. Fifth, the results of the AUC and Youden index showed that the discriminating ability of HKL angles for detecting large PIs was relatively low, as was the case for female patients. Such cases may require multiple measurements with different examiners to ensure the reliability of the PI classification tool. Sixth, standard procedures for training manuals should be prepared using various photos with a wide range of ages and/or multiple features of curvature of the spinal column. Further improvement is warranted to enhance the intra- and inter-rater reliability of the tools for practical use.

## 7. Conclusions

To explore effective measurement angles for PI classification, we determined the applicable HKL angle defined as the angle of two intersecting lines on the sagittal plane, the line connecting the most raised part of the buttock and central point of the knee, and the midthigh line (the femoral axis). This study revealed that the HKL angle could distinguish the size of PI with high/moderate discrimination ability. Furthermore, a quick, noninvasive assessment tool devised for PI classification using the cut-offs of the HKL angle indicated acceptable inter- and intra-rater reliability, enough for practical application. 

## Figures and Tables

**Table 1:** The results of sensitivity, specificity, and Youden index of the HKL angle C.

HKL Angle C	Cut-Off	Total (*n* = 125)			Male (*n* = 71)			Female (*n* = 54)		
		sen.	spec.	Y. I	sen.	spec.	Y. I	sen.	spec.	Y. I
S/ML	13.0	1.00	0.00	0.00	1.00	0.00	0.00	–	–	–
	14.5	1.00	0.04	0.04	1.00	0.05	0.05	1.00	0.00	0.00
	15.5	0.98	0.32	0.30	1.00	0.38	0.38	0.96	0.10	0.10
	16.5	0.97	0.36	0.33	1.00	0.57	0.43	–	–	–
	17.5	0.97	0.57	0.54	1.00	0.71	0.71	0.94	0.08	0.08
	18.5	0.91	0.79	0.69	0.98	0.81	0.79	0.83	0.71	0.54
	19.5	0.81	0.93	0.74	0.90	0.95	0.85	0.72	0.86	0.58
	20.5	0.70	0.96	0.66	0.76	0.95	0.71	0.63	1.00	0.63
	21.5	0.50	1.00	0.49	0.54	1.00	0.54	0.44	1.00	0.44
	22.5	0.32	1.00	0.32	0.32	1.00	0.32	0.33	1.00	0.33
	23.5	0.16	1.00	0.16	0.14	1.00	0.14	0.17	1.00	0.17
	24.5	0.09	1.00	0.08	0.08	1.00	0.08	0.09	1.00	0.09
	25.5	0.02	1.00	0.02	0.02	1.00	0.02	0.02	1.00	0.02
	26.5	0.01	1.00	0.01	0.00	1.00	0.00	–	–	–
	28.0	0.00	1.00	0.00	–	–	–	0.00	1.00	0.00
**HKL**	**Cut-off**	**Total (*n* = 125)**			**Male (*n* = 71)**			**Female (*n* = 54)**		
**angle C**		**sen.**	**spec.**	**Y. I**	**sen.**	**spec.**	**Y. I**	**sen.**	**spec.**	**Y. I**
SM/L	13.0	1.00	0.00	0.00	1.00	0.00	0.00	–	–	–
	14.5	1.00	0.01	0.01	1.00	0.02	0.02	1.00	0.00	0.00
	15.5	1.00	0.11	0.11	1.00	0.13	0.13	1.00	0.08	0.08
	16.5	1.00	0.13	0.13	1.00	0.15	0.15	–	–	–
	17.5	1.00	0.20	0.20	1.00	0.25	0.25	1.00	0.11	0.11
	18.5	1.00	0.32	0.32	1.00	0.30	0.30	1.00	0.36	0.36
	19.5	0.93	0.43	0.36	1.00	0.41	0.41	0.88	0.47	0.36
	20.5	0.89	0.55	0.44	1.00	0.53	0.53	0.82	0.58	0.41
	21.5	0.74	0.72	0.46	0.90	0.70	0.61	0.65	0.75	0.40
	22.5	0.59	0.84	0.44	0.80	0.87	0.67	0.47	0.81	0.28
	23.5	0.37	0.95	0.32	0.50	0.97	0.47	0.29	0.92	0.21
	24.5	0.19	0.97	0.15	0.30	0.98	0.28	0.12	0.94	0.06
	25.5	0.07	1.00	0.07	0.10	1.00	0.10	0.06	1.00	0.06
	26.5	0.04	1.00	0.04	0.00	1.00	0.00	–	–	–
	28.0	0.00	1.00	0.00	–	–	–	0.00	1.00	0.00

Note: sen., sensitivity; spec., specificity; Y. I, Youden index, S/ML, thresholds of HKL C angles discriminating between small (<42°) and more than medium (≥42°) pelvic incidence; SM/L, thresholds of HKL C angles discriminating between less than medium (<51°) and large (≥51°) pelvic incidence. Columns shaded in gray in the table mean out of criteria (unacceptable range of cut-off points); namely, sensitivity and specificity of >0.7.

**Table 2:** Results of usability metrics and intra-/inter-rater reliability of the PI classification tool using the HKL angle.

Sub. No.	Mean Time (s/photo)		Correct Rate (%)		Intrarater Reliability Cohen Kappa (95% CI)			Inter-Rater Fleiss’s Kappa (95% CI)	
	1st	2nd	1st	2nd	Total (*n* = 24)	Male (*n* = 14)	Female (*n* = 10)	1st	2nd
1	20.0	13.8	88	83	0.87 (0.72–1.00)	0.93 (0.77–1.00)	0.68 (0.34–1.00)	total 0.50 (0.47–0.53) male 0.43 (0.39–0.47) female 0.56 (0.46–0.68)	total 0.54 (0.51–0.57) male 0.50 (0.27–0.57) female 0.57 (0.51–0.73)
2	17.5	13.8	79	88	0.80 (0.61–0.99)	0.83 (0.58–1.00)	0.69 (0.38–1.00)		
3	13.1	12.9	83	71	0.82 (0.67–0.97)	0.74 (0.52–0.96)	1.00 (1.00–1.00)		
4	14.6	12.0	79	83	0.87 (0.71–1.00)	0.78 (0.68–0.89)	0.78 (0.39–1.00)		
5	19.6	17.1	67	83	0.75 (0.56–0.94)	0.75 (0.50–0.99)	0.69 (0.38–1.00)		
6	16.0	14.0	83	83	0.78 (0.60–0.97)	0.71 (0.48–0.95)	0.85 (0.63–1.00)		
7	13.2	12.6	83	71	0.67 (0.45–0.90)	0.58 (0.27–0.88)	0.80 (0.44–1.00)		
8	11.3	10.6	79	88	0.75 (0.57–0.94)	0.71 (0.42–0.99)	0.79 (0.52–1.00)		
9	12.5	11.3	75	75	0.83 (0.67–1.00)	0.70 (0.41–0.99)	1.00 (1.00–1.00)		
10	15.0	13.1	67	79	0.67 (0.45–0.88)	0.69 (0.43–0.95)	0.59 (0.29–0.90)		
11	24.6	18.3	71	67	0.82 (0.64–0.99)	0.91 (0.75–1.00)	0.63 (0.38–0.88)		
12	21.7	10.3	67	88	0.81 (0.66–0.97)	0.89 (0.74–1.00)	0.63 (0.38–0.88)		
13	26.3	19.8	83	83	0.77 (0.58–0.95)	0.83 (0.61–1.00)	0.65 (0.43–0.87)		
14	12.8	10.4	75	75	0.82 (0.65–0.99)	0.83 (0.62–1.00)	0.76 (0.42–1.00)		
mean (sd)	17.0 (4.7)	13.6 (2.9)	77.1 (6.9)	79.8 (6.9)	0.79 (0.61–0.96)	0.78 (0.59–0.97)	0.75 (0.50–0.97)		

Note: 95% CI, 95% confidence interval; sub., subject; s, second.

## Data Availability

The anonymized data presented in this study are available upon request from the corresponding author. The data are not publicly available due to privacy considerations or ethical challenges.
